# Effects of low-level laser photobiomodulation on the masticatory function and mandibular movements in adults with temporomandibular disorder: a systematic review with meta-analysis

**DOI:** 10.1590/2317-1782/20212021138

**Published:** 2022-01-31

**Authors:** Camila Fonsêca Guedes Pereira Máximo, Julyane Feitoza Coêlho, Silvia Damasceno Benevides, Giorvan Ânderson dos Santos Alves

**Affiliations:** 1 Programa Associado de Pós-graduação em Fonoaudiologia – PPgFon UFPB/UFRN, Universidade Federal da Paraíba – UFPB - João Pessoa (PB), Brasil.; 2 Programa de Pós-graduação em Linguística, Universidade Federal da Paraíba – UFPB - João Pessoa (PB), Brasil.; 3 Departamento de Fonoaudiologia, Universidade Federal da Paraíba – UFPB - João Pessoa (PB), Brasil.

**Keywords:** Low-Level Light Therapy,, Temporomandibular Joint Dysfunction Syndrome,, Mastication,, Mandible,, Systematic Review

## Abstract

**Purpose:**

To review the effects of low-level laser photobiomodulation on masticatory function and mandibular movements in adults with temporomandibular disorder.

**Research strategies:**

Search in PubMed, Web of Science, Scopus, EMBASE, Cochrane, LILACS, ScienceDirect, and Google Scholar, using the following descriptors: “temporomandibular joint disorders”, “low-level light therapy”, “low-level laser therapy”, “mastication”, and “mandible”.

**Selection criteria:**

Randomized clinical trials in adults with temporomandibular disorder, using low-level laser and assessing the mastication and mandibular movements.

**Data analysis:**

Firstly, the titles and abstracts of all retrieved studies were read. Then, only the studies selected in the first stage were read in full and assessed regarding eligibility. After the selection, the characteristics, methodological quality, and quality of evidence of the studies included in the review were analyzed. In the meta-analysis, the mean amplitude of mouth opening was considered as a measure of intervention effect.

**Results:**

The 10 articles included in the review had quite different results one from the other, especially regarding the amplitude of mouth opening, while the mastication was assessed in only one of them. Most studies had a high risk of bias, demonstrating a low methodological quality. Significantly higher results for photobiomodulation were identified in the six studies included in the meta-analysis.

**Conclusion:**

Due to the scarcity in the literature, there is not enough evidence of the effects of low-level laser photobiomodulation on mastication. As for the mandibular movements, this intervention presented significant results, particularly in the amplitude of mouth opening.

## INTRODUCTION

The temporomandibular disorder (TMD) is a set of dysfunctions involving the masticatory muscles, the temporomandibular joint (TMJ), and associated structures^(1)^. This disorder has a variety of causes, including predisposition, precursors, and attenuators, such as deleterious habits, occlusal changes, condyle-disc imbalance, stress, and anxiety^(2)^. Depending on its etiology and symptoms, TMD can be classified as myogenous, arthrogenous, or mixed^(2)^.

The most common TMD symptoms are joint noises (crepitation and clicking), otalgia, tinnitus, head and neck pain, headache, hyper- or hypofunction of the masticatory muscles, tooth sensitivity, mandibular deviations, limited mouth opening, impaired sleep, and emotional changes, thus diminishing the patients’ quality of life^(3,4)^.

This pathology has been significantly growing, affecting more women than men, occurring mostly between 20 and 50 years old^(5)^. Since its etiology is multifactorial, the treatment is carried out according to the signs and symptoms in each patient, always instructing them properly, as decreasing some habits may help the intervention^(2)^.

The treatments make use of less invasive or noninvasive procedures, such as medication therapy, orofacial myofunctional therapy, psychological treatment, interocclusal splint, acupuncture, electrostimulation, viscosupplementation, ultrasound therapy, and laser therapy. More invasive procedures are also used, as in the case of surgeries^(6)^. TMD therapy in the field of speech-language-hearing pathology is quite effective in the rehabilitation of the masticatory system and mandibular movements, using oral-motor function exercises and techniques to achieve a more adequate and balanced muscle functioning^(7)^.

The word laser is an acronym that stands for light amplification by stimulated emission of radiation. Better known as light therapy, phototherapy, or photobiomodulation (PBM), it is one of the oldest therapy methods manipulated by humans. It is classified into two types: high-power laser (which is ablative) and low-power laser (which is therapeutic)^(8)^.

PBM therapy is a non-pharmacological, painless, noninvasive treatment without side effects and whose main functions are analgesic, anti-inflammatory, and tissue regenerative. It transforms light energy into chemical energy, inducing metabolic, energetic, and functional changes and helping increase cell resistance and vitality^(9)^.

In other fields, such as dentistry and physical therapy, which have been using laser as a therapy technology for longer, there are many studies with scientific evidence of this resource in TMD^(9,10)^. Generally, though, the most studied outcomes are related to analgesic effects and mandibular movements^(11-20)^.

The pain and discomfort in TMD patients can have negative effects on the performance of the stomatognathic functions. A study in patients with moderate-to-severe chronic TMD identified, with functional and electromyographic assessment, significantly greater difficulty in mastication, worse orofacial scores, longer free mastication, unprecise muscle recruitment on the work and balance sides, lower symmetrical mastication rates, and increased patterned activity during the electromyographic test in comparison with healthy people^(21)^.

The analgesic and biomodulator effects of low-level laser (LLL) therapy, acting upon the algesic and inflammatory processes, can help ease these patients’ pain and discomfort, improve muscle performance and diminish the sensitivity of the masticatory muscles and other pain points. Thus, combined with speech-language-hearing therapy, this resource may increase the amplitude of mandibular movements, improve the masticatory function, and provide greater harmony in the stomatognathic system^(22)^.

Secondary studies that researched the evidence of LLL in TMD revealed the importance of PBM therapy to ease the pain and improve mandibular functioning. They also investigated the effects obtained in combining it with other interventions. The reviews that have been carried out until now have mostly approached functioning; hence, they do not cover the topic in-depth, generally considering it a secondary objective^(9,23,24)^.

Therefore, this study was developed to analyze the available evidence of the use of this resource in mandibular movements and masticatory function. These mutually related aspects are of central interest in speech-language-hearing intervention in the field of oral-motor function in cases of TMD. This review was written based on the Preferred Reporting Items for Systematic Reviews and Meta-Analyses (PRISMA)^(25)^ and registered in the International Prospective Register of Systematic Reviews (PROSPERO), under number CRD42020187091.

## PURPOSE

Hence, this study aimed to make a systematic review of the evidence of LLL PBM to investigate the effects of this technique on the masticatory function and mandibular movements in adults with TMD.

## RESEARCH STRATEGY

The search strategy was developed with the guidance of a librarian who worked in the originating institution, being adapted to each database and using their specific descriptors. The terms were selected from descriptors in PubMed’s Medical Subject Headings (MeSH) and EMBASE’s Emtree, considering the pathology researched, the intervention, and the outcomes included in the review.

The search strategy was simplified, encompassing the main index terms available in the vocabulary (thesaurus) of the databases. Previous tests of the search strategy revealed that these were enough to retrieve the eligible studies.

The search was conducted in PubMed, LILACS (via Virtual Health Library), Web of Science, Cochrane Library, EMBASE, Scopus, and ScienceDirect, besides an additional search for gray literature on Google Scholar and Open Grey. The reference lists in the articles included in this study were also analyzed to include any additional references that had not been identified in the databases. The Brazilian Registry of Clinical Trials was also surveyed to obtain further information on the studies that were included and identify possible studies in the process of being published. The search strategies used in the databases are shown in Table 1.

**Table 1:** Search strategies used in the databases

**Strategy**	**Database**
Search: ((“temporomandibular joint disorders”) AND (“low-level light therapy”)) AND (mastication) (“temporomandibular joint disorders”[All Fields] AND “low-level light therapy”[All Fields]) AND (((((((“masticated”[All Fields] OR “masticates”[All Fields]) OR “masticating”[All Fields]) OR “mastication”[MeSH Terms]) OR “mastication”[All Fields]) OR “masticate”[All Fields]) OR “mastication”[All Fields]) OR “masticator”[All Fields]) OR (((“mandible”[MeSH Terms] OR “mandible”[All Fields]) OR “mandibles”[All Fields]) OR “mandible s”[All Fields])	PubMed
((“temporomandibular joint disorders”) AND “low-level light therapy”) AND “mastication” OR “mandible” “([Quick search])- Quick Search	EMBASE
(tw:(“temporomandibular joint disorders”)) AND (tw:(“low-level light therapy”)) AND (tw:(mastication)) OR (tw:(mandible)) ([Title, abstract, and topic])	LILACS
“temporomandibular joint disorder” AND “Low-level light therapy” AND mastication OR mandible OR mastication*) ([All fields])	Cochrane Library
ALL= (Temporomandibular Joint disorder* AND Low-level laser therapy* AND mastication* OR mandible*) ([All fields])	Web of Science
ALL (“temporomandibular joint disorders”) AND ALL (“low-level light therapy”) AND ALL (mastication) OR ALL (mandible)) ([All fields])	Scopus
“temporomandibular joint disorders” AND “low-level light therapy” OR “low-level laser therapy” AND “mastication” OR “mandible” AND “Randomized Controlled Trial”	Google Scholar
“temporomandibular joint disorders” AND “low-level light therapy” OR “low-level laser therapy” AND “mastication” OR “mandible” AND “Randomized Controlled Trial”	ScienceDirect

The references were managed with the EndNote online software to remove the duplicate ones. All the database surveys took place between May 18 and 20, 2020, and were updated on September 16, 2020.

## SELECTION CRITERIA

The research question used to develop this research was based on the PICOT strategy, in which P (population) was adults with TMD; I (intervention) was LLL PBM; C (comparison) was other interventions or absence of interventions; O (outcomes) was masticatory function and/or mandibular movement measures; T (types of studies) was the randomized clinical trials. Thus, the research question was established as follows: “What are the effects of LLL on the performance of the masticatory function and mandibular movements in TMD patients, compared with other interventions or the absence of other interventions?”.

Original articles designed as randomized clinical trials were eligible without restrictions of time or language. The studies involved adults aged 18 to 60 years old, clinically diagnosed with TMD, using LLL intervention, and assessing the masticatory function and/or mandibular movements. The articles with other designs, with either children or older adults, whose text was not fully available, with other comorbidities, or with other treatments combined and applied simultaneously with laser were excluded.

These aspects were selected based on the age range used in most studies in the field, considering both the development of the stomatognathic system and the changes resulting from the natural aging process, as they might influence the measurement of the intervention effects. The presence of other comorbidities and other treatments applied simultaneously with LLL would likewise prevent a more precise analysis of the results. The main outcomes were chosen because of their clinical relevance in speech-language-hearing therapy in TMD cases.

## DATA ANALYSIS

The studies were selected in two stages, independently carried out by the same investigators. Firstly, the titles and abstracts of all studies were read, excluding the ones that did not meet the previously established eligibility criteria. In the second stage, the texts were read in full. In both stages, there was a strong interrater agreement, verified with Cohen’s kappa coefficient. The disagreements were discussed between the authors in both stages of the review process. When they still did not agree, a third reviewer got involved in the process, independently reading the studies and judging their eligibility.

In the data extraction phase, the information was likewise collected independently by the two reviewers. A specific instrument was developed for this stage, and the data were checked in a consensus meeting. The data of the selected articles were tabulated based on some characteristics: author, country, sample, objective, intervention parameters, use of the Research Diagnostic Criteria for Temporomandibular Disorders (RDC/TMD), type of intervention, outcomes, results, and conclusion. When their data were incomplete or absent, the reviewers contacted the authors via the corresponding e-mail to obtain all the necessary information.

The methodological quality of the studies was individually and independently assessed by two reviewers, following the Cochrane risk-of-bias tool for randomized trials (RoB 2)^(26)^. The analysis of the quality of evidence was made with the Grading of Recommendations Assessment, Development and Evaluation (GRADE)^(27)^.

The measure of intervention effect considered for the meta-analysis was the mean amplitude of mouth opening because it was verified as the main parameter used to assess mandibular movements in most studies included in the review. Only six studies presented in the results the mean, standard deviation, and the number of participants in each group, contributing directly to the synthesis. As for the assessment of the masticatory function, only one study considered this outcome. The measures used for the meta-analysis were the mean and standard deviation, with the inverse variance method, in the R statistical software.

## RESULTS

**Figure 1:**
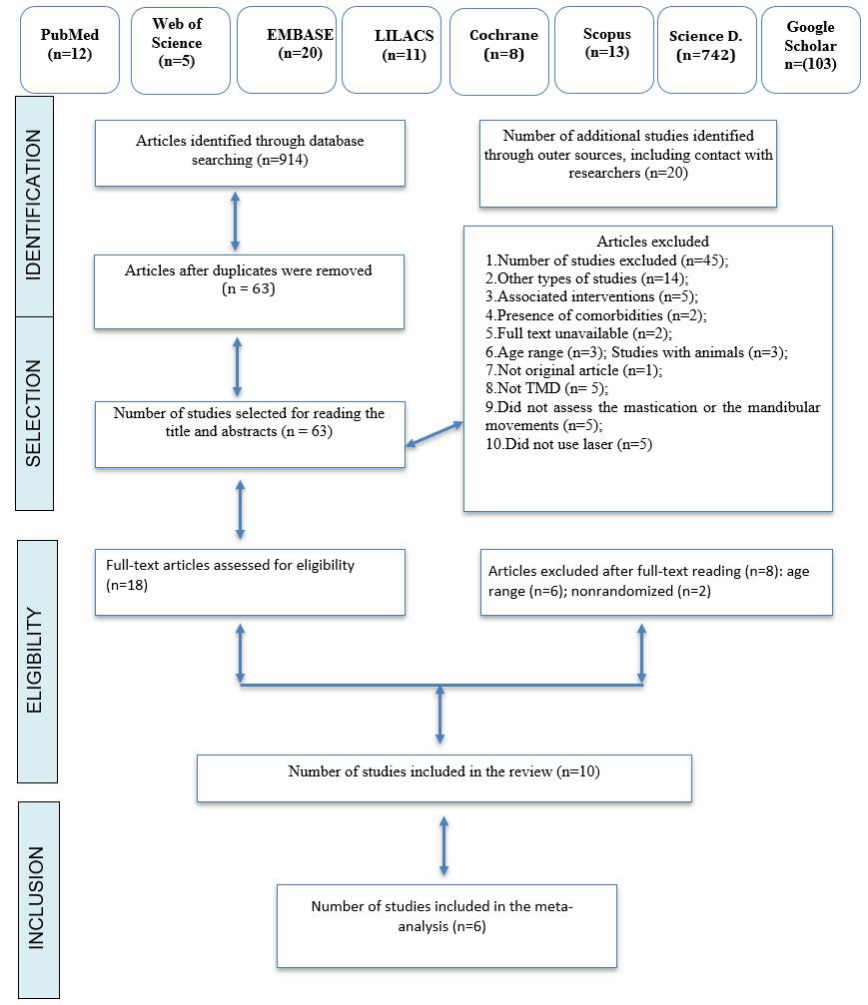
Flowchart of the study search and selection process

The study search and selection process is presented in detail in Figure 1.

Caption: MD = Mean difference; SD= Standard deviation

Five out of the 10 studies selected are Brazilian^(11-15)^, and five are international^(16-20)^. Four of the national ones are from the state of São Paulo^(11,13-15)^, and one is from Rio Grande do Sul^(12)^. As for the other articles, three are from Istanbul, in Turkey^(16,18,20)^, and two from Tehran, in Iran^(17,19)^.

The sample size ranged from 15^(12)^ to 82 participants^(15)^. Concerning the protocol to diagnose the TMD, eight used the RDC/TMD^(11,14-20)^, and two did not report the instrument used for the diagnosis^(12,13)^.

Regarding the masticatory function and the mandibular movements, six studies approached the amplitude of mouth opening alone as one of the outcomes^(12-14,16,17,19)^, three analyzed the protrusive movements, opening movements, and lateral mandibular excursions^(11,18,20)^, and only one approached the masticatory function^(15)^. The characteristics, main outcomes, and conclusions of the studies included in the review are shown in detail in Chart 1.

**Chart 1:** Characteristics of the studies included in the review, main results, and conclusions

**Author**	**Country**	**Sample (n)**	**Objective**	**RDC/TMD**	**Intervention parameters**	**Type of intervention**	**Outcomes**	**Results**	**Conclusion**
Da Silva et al.^(11)^	Brazil	45	To assess the effects of LLL on subjects with intra-articular TMD and quantify and compare the severity of the signs and symptoms before, during, and after applying the laser.	Yes	A total of 10 sessions were conducted over 5 weeks, twice a week. The device energy during the applications was 70 mW, the application time varied according to the point, and the wavelength was 780 nm. For 52.5 J/cm^2^, the application time was 30 seconds and 60 seconds for 105.0 J/cm^2^. The laser was applied continuously on 5 condylar points, 3 on the masseter, and 3 on the temporal.	Two different laser doses and a placebo.	Mandibular movements and painful symptoms evoked with muscle palpation.	The analysis of variance showed statistically significant differences between the groups, with a level of 1% between doses, (except for the protrusion variables: significant difference of 5%; and opening: not significant), as well as between the assessments (except for laterality – 5% statistical difference.	This study concluded that the LLL increased the amplitude of the mandibular movements.
De Carli et al.^(12)^	Brazil	15	To compare the use of LLL and botulinum toxin to treat myofascial pain and verify whether they change the mouth opening in patients with TMD.	Not informed	Seven applications were made with 48-hour intervals in-between the applications. The wavelength was 830 nm, with a dose of 80 J/cm^2^ and a power of 100 Mw. It was applied on 2 points of the masseter and 1 on the temporal.	Laser and type A botulinum toxin.	The amplitude of mouth opening and intensity of the pain.	Regarding the amplitude of mouth opening, no statistically significant difference was found between the laser group and the toxin group, considering that neither group had a significant increase during the treatment (p = 0.272).	The two therapies did not result in statistically significant improvement in the amplitude of mouth opening.
Kato et al.^(13)^	Brazil	18	To compare the efficacy of transcutaneous electrical nerve stimulation (TENS) and LLL therapy to treat patients with chronic myogenous TMD.	Not informed	A total of 10 sessions were conducted over 4 weeks, 3 times a week, with a wavelength of 830 to 904 nm, the output of 4 J/cm^2^, and power of 100 mW, with sweep movements, for 9 minutes on each side of the face.	Laser and TENS	The amplitude of mouth opening and intensity of the pain.	The results showed an increase in the amplitude of mouth opening in both groups (p < 0.05). There was a significant improvement in the amplitude of mouth opening (initial mean of 42.5 mm – 43 mm in the laser group and 42 mm in the TENS group – and final mean of 47.4 mm – 47.6 and 47.2 mm in the laser and TENS groups, respectively. The ANOVA showed that there was no statistically significant difference between the groups, including the immediate effect (p = 0.860 and p = 0.091, respectively). However, there was a significant difference between the sessions (p<0.0010).	Both therapies were effective in decreasing the TMD signs and symptoms, but the immediate effect was not significant, and the cumulative effect may have been responsible for this improvement.
Kogawa et al.^(14)^	Brazil	19	To assess the effectiveness of LLL and microelectric neurostimulation (MENS) to treat TMD patients.	Yes	The laser therapy was conducted 3 times a week, totaling 10 sessions, with a wavelength of 830 to 904 nm, the output of 4 J/cm^2^, and power of 100 mW.	Laser and MENS	The amplitude of mouth opening, muscle and TMJ palpation, and visual analog scale.	The results showed an increase in the amplitude of mouth opening. The initial and final means of amplitude in the laser group were 46.3 mm and 49.4 mm, respectively, while in the microelectric neurostimulation (MENS) group, it was 46.3 mm and 44 mm, respectively. There was no significant difference between the groups.	The conclusion was that both therapies (laser and MENS) are effective to treat myogenous TMD, but caution in result analysis is recommended, due to the self-limiting aspect of TMD. The amplitude of mouth opening improved in both groups.
Machado et al.^(15)^	Brazil	82	To investigate the efficacy of combining oral myotherapy exercises and LLL, in comparison with oral myofunctional therapy protocol, with LLL therapy alone, and placebo combined with exercises. To verify the effects of each program immediately after the treatment and in long-term follow-up.	Yes	Twelve weekly sessions for 60 days, and then every 2 weeks. Continuous emission at 780 nm wavelength, 60 mW power, and energy density varying around 60 +- 1 J/cm^2^. It was applied on 5 TMJ points and on the masseter and temporal muscles.	Laser + oral myotherapy exercises; laser alone; placebo + oral myotherapy exercises and complete oral myofunctional therapy protocol.	Self-assessment of TMD severity, pain with palpation, the subjective intensity of the pain, orofacial myofunctional status (appearance/posture, mobility, and performance of the stomatognathic functions).	The results of the orofacial myofunctional evaluation protocol with scores were lower than in the placebo group, without statistically significant results between the groups.	The LLL combined with oral myotherapy exercises was more effective than LLL alone, decreasing the TMD signs and symptoms and improving the mandibular movements.
Öz et al.^(16)^	Turkey	40	To assess the efficiency of the laser and compare it with occlusal splints to treat myofascial pain.	Yes	A total of 10 sessions were conducted, twice a week. The wavelength was infrared (820 nm), with a dose of 3 J/cm^2^, an output power of 300 mW, lasting 10 s on each point. The laser was precisely and continuously applied to the trigger points.	Laser and interocclusal splints.	Functional examination, finding the pressure pain threshold and amplitude of mouth opening.	Both groups had statistically significant improvements in the vertical mandibular movements after the treatment, and no significant differences were identified between the groups.	The laser was as effective as the interocclusal splints, and it can be an alternative treatment, as it is noninvasive and non-pharmacological.
Seifi et al.^(17)^	Iran	40	To assess the effect of LLL therapy and TENS on TMD.	Yes	Four half-hour sessions were conducted per week. The wavelength was 810 nm, with a continuous power of 0.5 W, for 60 s.	Laser, laser placebo, TENS, and TENS placebo.	Sensitivity of the masticatory muscles and amplitude of mouth opening.	The amplitude of mouth opening improved with time. However, it did not remain after 1 month in relation to the baseline (p=0.192). There were no significant differences between the LLL and TENS groups (p=0.820) in any of the stages. No statistically significant differences were found between the placebos (p=0.738). Mouth opening was significant in the laser and TENS groups and greater than in the placebo groups (p=0.002), though not in the 1-month follow-up (p=0.692).	The effects of laser and TENS can improve the TMD signs and symptoms in the short run and can be physical modalities of complementary and supplemental alternatives in TMD.
Gökçen-Röhlig et al.^(18)^	Turkey	40	To investigate the efficacy of LLL with 820 nm, 3 J/cm^2^, 300 Mw output power to treat myogenous TMD.	Yes	A total of 10 laser sessions were conducted daily for 3 weeks. The wavelength was infrared (820 nm), with a dose of 8 J/cm^2^ and power of 300 mW, for 10 s. It was continuously applied on 3 points of the masseter, 1 on the temporal, and 1 on the sternocleidomastoid muscle.	Laser and placebo	pressure pain threshold, visual analog scale, and mandibular mobility.	The laser group had statistically significant improvements in the vertical mandibular movements and lateral excursions.	The effects of laser are better than the results of the placebo group.
Madani et al.^(19)^	Iran	45	To investigate the efficacy of LLL to treat osteoarthritis and TMD.	Yes	A total of 12 sessions were conducted 3 times a week, for 4 weeks. The laser was applied with contact, with a power of 50 mW, 6 J per point, 3.4 J/cm^2^ on 4 points of the TMJ, 3 on the masseter, 3 on the temporal, and on the attachment of the internal pterygoid muscle.	Laser, laser acupuncture, and placebo	Degree of pain, the amplitude of mouth opening, and joint noises.	After 12 sessions of laser application, the mean amplitude of mouth opening increased from 29.2 mm to 31.7 mm (an 8% increase) in the laser group. In the placebo treatment, it increased from 23.5 mm to 24.7 mm (a 5% increase). The analysis did not show a statistically significant difference between the groups of study or between the different assessment times in each group.	The laser and placebo group did not significantly improve the amplitude of mouth opening.
Sancakli et al.^(20)^	Turkey	30	To assess the effect of applying LLL in the main pain points in patients with chronic masticatory muscle pain (myofascial pain).	Yes	A total of 12 sessions were conducted 3 times a week, for 1 month. The wavelength was infrared (820 nm), with a dose of 3 J/cm^2^ and output power of 300 mW for 10 s. It was applied on the main pain points and 3 points of the masseter and temporal muscles.	Laser on the main pain points and preestablished points.	Mandibular mobility (amplitude, lateral excursions, protrusion), pressure pain threshold, pressure measure, and subjective measure of the pain.	The vertical mandibular movements, lateral excursions, and protrusion improved significantly in the LLL groups.	The LLL had positive effects on the mandibular movements due to its analgesic and myorelaxant effects. There was no difference between the application on the pain and preestablished points.

The studies that had significant results in the amplitude of mandibular movement showed that the higher the dose used, the more immediate and expressive the effects. The measures were taken between the first, fifth, tenth, and twelfth sessions, even up to one month after the laser intervention.

Some studies pointed out that the results of the amplitude of mouth opening had not been statistically significant between the groups^(12-14,16,17,19)^. Concerning the vertical, lateral excursion, and protrusive movements, three articles^(11,18,20)^ showed statistically significant results.

Only one of the studies assessed the masticatory function, demonstrating that LLL PBM therapy in combination with oral myofunctional exercises is more effective than LLL alone, diminishing the signs and symptoms of TMD and improving the mandibular movements. In the study in question, the overall mobility and function score results indicated lower results in the group treated only with laser therapy, with significant differences between the groups^(15)^.

Nine out of the 10 studies in this review were grouped for quantitative analysis of the results because they presented the amplitude measure of mouth opening. However, only six of them could be used in the meta-analysis. The studies were rather different from one another, especially regarding maximum mouth amplitude.

**Figure 2:**
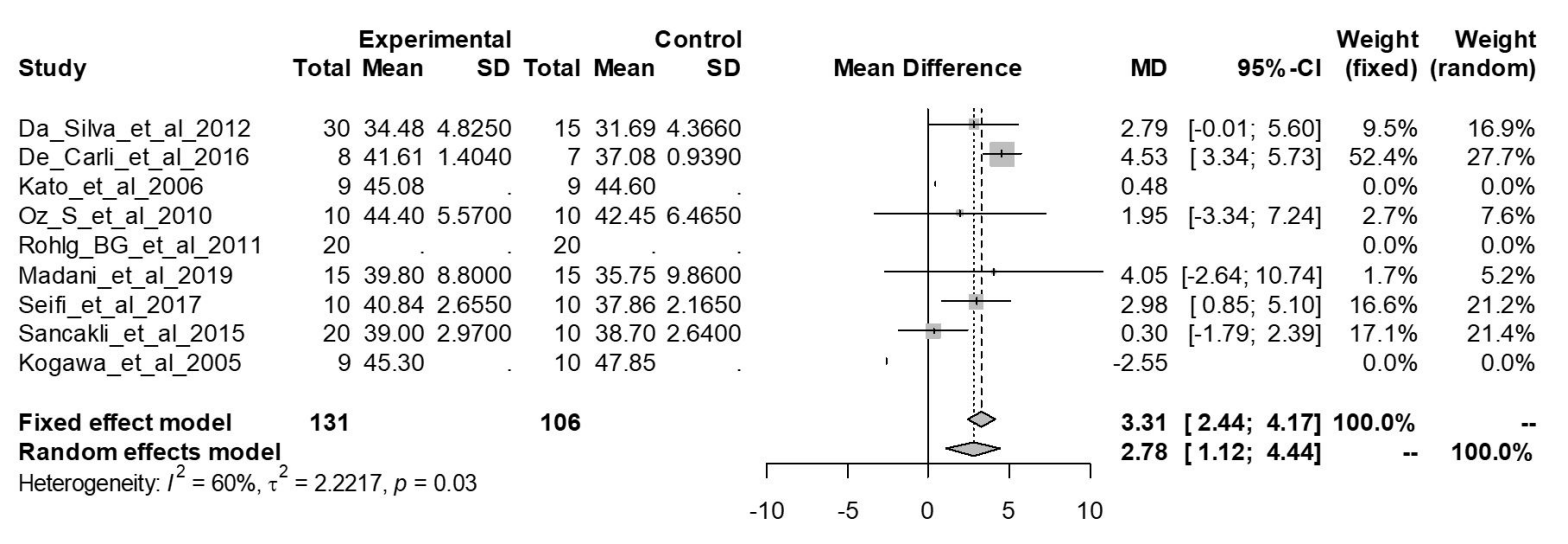
Forest Plot of the meta-analysis of the studies

In the quantitative analysis, the diamond at the end of the plot reflects the combination of results. It is on the right side and did not touch the axis, which means the treatment was better in the experimental group – i.e., it had significant results. In the difference of means column, the value reveals that the experimental group was better – 2.78 points on a scale from 0 to 100 in the random models. Concerning the heterogeneity between the studies, the I^2^ was 60%, indicating moderate heterogeneity. The quantitative synthesis is shown in detail in Figure 2.

The studies included in the review had a quite heterogeneous methodology. Five articles^(11,13,14,17,18)^ were generally classified with a high risk of bias, two were classified with some concern^(16,20)^, and three, with a low risk^(12,15,19)^ in the quality assessment.

**Figure 3:**
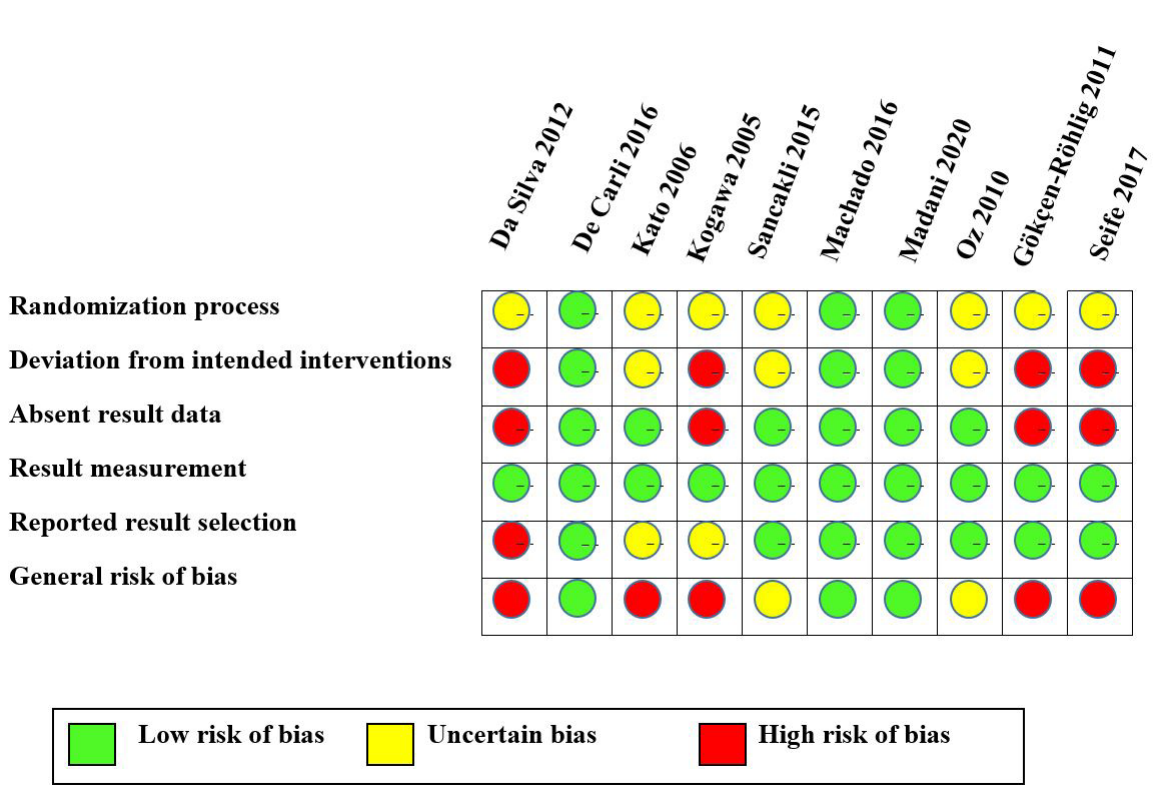
General classification and categorization of the quality of the studies included in the review

The main methodological limitations in the studies were related to unreported information on generating random sequences, allocation concealment, and participants’ blinding, as shown in Figure 3.

Since the review used outcomes from randomized clinical trials, the assessment of the quality of evidence began with the maximum score, which was then decreased in some parameters, as shown in Chart 2.

**Chart 2:** Quality of evidence (GRADE)

**Summary of the Results**								
**LLL photobiomodulation compared with placebo or other interventions for temporomandibular disorder**								
**Patient or population**: Temporomandibular disorder **Context**: Mandibular movements and masticatory function **Intervention**: LLL photobiomodulation **Comparison**: Placebo or other interventions								
Outcome No. of participants (studies)	Relative effect (95% CI)	**Potential absolute effects (95% CI)**					Certainty	What happens
		Without photobiomodulation with LLL	With photobiomodulation with LLL		**Difference**			
Mandibular movements assessed with: mouth opening No. of participants: 160 (6 RCTs)	-	The mean of the mandibular movements was **37.25** mm		The mean of the mandibular movements was **40.03** mm		DM **2.78 mm** higher (1.12 higher for 4.44 higher)	⨁◯◯◯ VERY LOW ^a,b,c^	The LLL photobiomodulation can increase/have little or no effect on the mandibular movements, but the evidence is very uncertain.
Masticatory function assessed with OMES protocol No. of participants: 39 (1 RCT)	The study presented only the analysis of the total scores of the stomatognathic functions, identifying that the group with LLL photobiomodulation did not have significant results after the treatment. On the other hand, the comparison between the various intervention groups identified significant results.						⨁⨁⨁⨁ HIGH	The study had a high methodological quality. However, it would be necessary to analyze the mastication, specifically, as well as the combination with other studies. Thus, there is not enough evidence of the effects of LLL photobiomodulation on the masticatory function.
**Levels of evidence of the GRADE Working Group** **High certainty**: We are very confident that the actual effect is close to the estimated effect. **Moderate certainty**: We are moderately confident about the estimated effect; the actual effect is probably close to the estimated effect, but it may be substantially different. **Low certainty**: We have limited confidence in the estimated effect; the actual effect may be substantially different from the estimated effect. **Very low certainty**: We are very little confident about the estimated effect; the actual effect is probably substantially different from the estimated effect.								
**Explanations** a. The studies had many methodological limitations regarding the outcome assessed. Three of the six studies had a high risk of bias – one with an uncertain risk and two with a low risk –, which helped lower the quality of evidence. Some of the aspects identified were the lack of participant allocation concealment, lack of blinding, and lack of information on losses to follow-up. b. The comparison of the studies identified the presence of inconsistency, as various methodologies were used for the same outcome, with different intervention parameters and results. This led to considerable heterogeneity, also verified in the statistical analysis, which lowered the quality of evidence. c. Some studies were imprecise regarding the amplitude of the 95% confidence interval, decreasing the confidence in the estimated effects.								

Caption: CI = confidence interval; DM = difference of means

There were no significant results in the comparison between laser with type A botulinum toxin interventions^(12)^ and microelectric neurostimulation (MENS)^(14)^ regarding mandibular movements. On the other hand, a study compared transcutaneous electrical nerve stimulation (TENS) with LLL and reported the efficacy of both therapies, with a difference between the groups only in the cumulative effect^(13)^. In another one, no significant differences were found between the LLL and TENS groups in any of the stages^(17)^.

The comparison between two different LLL modalities or between LLL and a placebo group^(11,18,19)^ revealed significant and higher results for the groups submitted to the intervention. The paper that compared the laser with orofacial myofunctional therapy^(15)^ identified great results from this therapy alone. However, it was not combined with LLL therapy, which may be an alternative to obtain more significant results.

An important aspect to consider is the dose used. Some studies used a low dose (between 1.5 J and 3 J), which, given the objectives, may have prevented significance. Hence, a higher dose would be necessary. Considering the results, there is great inconsistency and methodological flaws between these studies, which deemed five studies with a high risk of bias, decreasing their quality of evidence and reliability.

The laser protocol used in each study also varied greatly. The number of sessions in the studies was balanced in 10 to 12, which is the advocated in the literature for being considered the adequate number of sessions to obtain significant results. As for the frequency of sessions, it varied between once a week, every day for 4 weeks, for 5 weeks, or every 2 weeks.

The wavelength ranged from 780 nm to 904 nm, revealing that all studies used infrared wavelength. The greatest difference between the studies was the dose, which ranged from 1.5 J/cm^2^ to 105.0 J/cm^2^, depending on the equipment they used. This shows how heterogeneous the studies were. Future clinical trials with laser must choose more homogeneous protocols, with greater methodological rigor, for the results to have more reliable evidence.

Five out of the 10 studies are Brazilian^(11-15)^, which shows that Brazil is strong in publications in the field of PBM and TMD. Moreover, three of these studies are from the same research group^(13-15)^. Almost all clinical trials are from fields such as physical therapy or dentistry, whereas only one article^(15)^ had a speech-language-hearing therapist among its authors – which is also the only one that analyzed the masticatory function. This may have occurred because these sciences have been using the laser for longer, while in speech-language-hearing pathology its use was regulated only in 2019, with Resolution no. 541^(28)^, and it has been applied in clinical practice only recently.

Such aspects show the need for further research on the masticatory function and mandibular movements on the part of these professionals, as they are essential in TMD therapy. We currently have positive clinical findings available, but further scientific evidence is necessary to recommend the therapeutic choice and decision-making for using this resource, instead of or in combination with the other ones already available in the field.

Since the laser can both stimulate and inhibit the tissue response, it can help develop functions that were changed in people with TMD, including mastication, which has a considerable impact on this pathology^(22)^. It must be highlighted that, in the speech-language-hearing clinic, this technology must not be used in place of consistent, highly relevant therapies in the field, but rather as a complementary and alternative intervention to speed the treatment process. Thus, the intervention must be directed and individualized, integrating the various approaches involved in the care for people with TMD and considering the different speech-language-hearing and dental aspects involved in rehabilitating this function.

Given the above, some clinical implications stand out in this study. Intervention protocols evidently must be developed to better standardize important parameters, such as the dosímetry and the number and frequency of sessions, to obtain effective therapeutic results.

This review has some contributions, as it points out the main parameters and their results currently approached in the scientific literature in the field. Moreover, it highlights the effects obtained with laser in comparison with other interventions, thus making the speech-language-hearing therapists’ clinical practice easier in this field, in terms of choosing the best therapeutic approach to reach the desired objective.

Another important aspect is that the most recurrent measure in the literature, as both the main and secondary outcome, was the amplitude of mouth opening. Hence, this parameter measure is greatly important to quantify the results obtained in the laser intervention. Nevertheless, more robust assessments with broader criteria to analyze the various mandibular movements are indispensable.

Some limitations in this systematic review must be pointed out. The analysis of the studies revealed considerable variability. This may be due to the characteristics of each study, which applied rather diverging methodologies (sample size, type of intervention, power, energy dose, time of application, etc.). Thus, even though there are some positive effects regarding the efficacy of laser on TMD, the diversity of methodological parameters interfere with the conclusions obtained in each study, whose results are different from and conflicting with one another.

Besides the methodological differences found between the studies, they had a low quality of evidence, with a considerable bias in most studies. Moreover, the studies lacked some data, making it difficult to obtain information for a quantitative synthesis that would include all the results, enabling a broader analysis.

Therefore, this study verified that LLL PBM did not provide evidence of the effect of LLL on the masticatory function, although it demonstrated beneficial effects in terms of increasing the amplitude of the mandibular movements. The LLL therapy had positive impacts on the increase of the amplitude of mouth opening, with better results than the other interventions or the absence of treatment, as demonstrated in the meta-analysis.

Further clinical trials are needed, with more homogeneous, high-quality protocols, to find new clinical approaches and scientific evidence that can be replicated, especially in the field of speech-language-hearing pathology, which had few studies focused on the masticatory function.

## CONCLUSION

This study verified a scarcity in the literature regarding the masticatory function, as only one study analyzed this variable. Hence, the information available was not enough to analyze the effects of the LLL PBM therapy on this function. As for the mandibular movements, the methodological parameters and intended outcomes vary widely from one piece of research to another. In the intervention groups, the LLL PBM had significant results, which is made evident in the quantitative synthesis of the main outcome of the amplitude of mouth opening.

## References

[1] Leeuw R (2010). Dor orofacial: guia de avaliação, diagnóstico e tratamento..

[2] Gil-Martínez A, Paris-Alemany A, López-de-Uralde-Villanueva I, La Touche R (2018). Management of pain in patients with temporomandibular disorder (TMD): challenges and solutions. J Pain Res.

[3] Melchior MO, Machado BC, Magri LV, Mazzetto MO (2016). Effect of speech-language therapy after low-level laser therapy in patients with TMD: a descriptive study. CoDAS.

[4] Durham J, Newton-John TR, Zakrzewska JM (2015). Temporomandibular disorders. BMJ.

[5] Piccin CF, Pozzebon D, Chiodelli L, Boufleus J, Pasinato F, Corrêa EC (2016). Clinical and psychosocial aspects assessed by the research diagnostic criteria for temporomandibular disorder. Rev CEFAC.

[6] Sassi FC, Silva AP, Santos RKS, Andrade CRF (2018). Oral motor rehabilitation for temporomandibular joint disorders: a systematic review. Audiol Commun Res.

[7] Felício CM, Melchior MO, Silva MA (2010). Effects of orofacial myofunctional therapy on temporomandibular disorders. Journal Cranio Pract.

[8] Catão MH, Oliveira PS, Costa RO, Carneiro VS (2013). Evaluation of the efficacy of low-level laser therapy (lllt) in the treatment of temporomandibular disorders: a randomized clinical trial. Rev CEFAC.

[9] Frare JC, Nicolau RA (2008). Clinical analysis of the effect of laser photobiomodulation (GaAs – 904 nm) on temporomandibular joint dysfunction. Rev Bras Fisioter.

[10] Vivan CL (2019). O efeito da terapia com fotobiomodulação na dor, na qualidade de vida e na percepção da limitação funcional de indivíduos com disfunção temporomandibular: resultados preliminares.

[11] Silva MA, Botelho AL, Turim CV, Silva AM (2012). Low level laser therapy as an adjunctive technique in the management of temporomandibular disorders. Cranio.

[12] De Carli BMG, Magro AKD, Souza-Silva BN, Matos FS, De Carli JP, Paranhos LR (2016). The effect of laser and botulinum toxin in the treatment of myofascial pain and mouth opening: a randomized clinical trial. J Photochem Photobiol B.

[13] Kato MT, Kogawa EM, Santos CN, Conti PC (2006). Tens and low-level laser therapy in the management of temporomandibular disorders. J Appl Oral Sci.

[14] Kogawa EM, Kato MT, Santos CN, Conti PC (2005). Evaluation of the efficacy of low-level laser therapy (LLLT) and the microelectric neurostimulation (MENS) in the treatment of myogenic temporomandibular disorders: a randomized clinical trial. J Appl Oral Sci.

[15] Machado BCZ, Mazzetto MO, Da Silva MA, De Felício CM (2016). Effects of oral motor exercises and laser therapy on chronic temporomandibular disorders: a randomized study with follow-up. Lasers Med Sci.

[16] Öz S, Gökçen-Röhlig B, Saruhanoglu A, Tuncer EB (2010). Management of Myofascial Pain: Low-Level Laser Therapy Versus Occlusal Splints. J Craniofac Surg.

[17] Seifi M, Ebadifar A, Kabiri S, Badiee MR, Abdolazimi Z, Amdjadi P (2017). Comparative effectiveness of Low Level Laser therapy and Transcutaneous Electric Nerve Stimulation on Temporomandibular Joint Disorders. J Lasers Med Sci.

[18] Gökçen-Röhlig B, Kipirdi S, Meriç U, Capan N, Keskin H (2011). Masticatory muscle pain and low-level laser therapy: a double-blind and placebo-controlled study. Turk J Phys Med Rehab.

[19] Madani A, Ahrari F, Fallahrastegar A, Daghestani N (2020). A randomized clinical trial comparing the efficacy of low-level laser therapy (LLLT) and laser acupuncture therapy (LAT) in patients with temporomandibular disorders. Lasers Med Sci.

[20] Sancakli E, Gökçen-Röhlıg B, Balık A, Öngül D, Kıpırdı S, Keskın H (2015). Early results of low-level laser application for masticatory muscle pain: a double-blind randomized clinical study. BMC Oral Health.

[21] Ferreira CLP, Machado BCZ, Borges CGP, Silva MAMR, Sforza C, Felício CM (2014). Impaired orofacial motor functions on chronic temporomandibular disorders. J Electromyogr Kinesiol.

[22] Gomes FC, Schapochnik A (2017). The therapeutic use of low intensity laser (LLLT) in some diseases and its relation to the performance in speech therapy. Distúrb Comun.

[23] Xu GZ, Jia J, Jin L, Li JH, Wang ZY, Cao DY (2018). Low-level laser therapy for temporomandibular disorders: a systematic review with meta-analysis. Pain Res Manag.

[24] Santos N, Cavalcante J, Silva T, Santos S, Fernandes E, Leitão A (2020). Low-power laser use for tratment of temporomandibular muscle dysfunction: a systematic review. Braz J Health Rev..

[25] Page MJ, McKenzie JE, Bossuyt PM, Boutron I, Hoffmann TC, Mulrow CD (2021). The PRISMA 2020 statement: an updated guideline for reporting systematic reviews. BMJ.

[26] Higgins JPT, Savovic J, Page MJ, Sterne JAC (2019). Revised Cochrane risk-of-bias tool for randomized trials (RoB 2) Short Version (CRIBSHEET)..

[27] Grade Working Group (2014). The grading of recommendations assessment, development and evaluation.

[28] Brasil (2019). Resolução CFFa nº 541, de 15 de março de 2019..

