# Supplementary material for: Effects of low-level laser photobiomodulation on the masticatory function and mandibular movements in adults with temporomandibular disorder: a systematic review with meta-analysis
Source: Codas. 2022 Jan 31;34(3):e20210138. doi: 10.1590/2317-1782/20212021138 (PMC9769431; doi:10.1590/2317-1782/20212021138)
Supplement: Translation

# Translation: Fotobiomodulação com laser de baixa potência na função mastigatória e nos movimentos mandibulares em adultos com disfunção temporomandibular: revisão sistemática com metanálise

**Authors:** Camila Fonsêca Guedes Pereira Máximo, Julyane Feitoza Coêlho, Silvia Damasceno Benevides, Giorvan Ânderson dos Santos Alves

**Affiliations:** 1 Programa Associado de Pós-graduação em Fonoaudiologia – PPgFon UFPB/UFRN, Universidade Federal da Paraíba – UFPB - João Pessoa (PB), Brasil.; 2 Programa de Pós-graduação em Linguística, Universidade Federal da Paraíba – UFPB - João Pessoa (PB), Brasil.; 3 Departamento de Fonoaudiologia, Universidade Federal da Paraíba – UFPB - João Pessoa (PB), Brasil.

**Keywords:** Terapia com Luz de Baixa Intensidade,, Síndrome da Disfunção da Articulação Temporomandibular,, Mastigação,, Mandíbula,, Revisão Sistemática

## Abstract

**Objetivo:**

Revisar os efeitos da Fotobiomodulação com Laser de Baixa Potência na função mastigatória e nos movimentos mandibulares, em adultos com Disfunção Temporomandibular.

**Estratégia de pesquisa:**

Busca nas bases de dados PubMed, Web of Science, Scopus, Embase, Cochrane, Lilacs, Science Direct e Google Scholar, utilizando os descritores: “temporomandibular joint disorders”, “low level light therapy”, “low level laser therapy”, “mastication” e “mandible”.

**Critérios de seleção:**

Ensaios clínicos randomizados envolvendo adultos com Disfunção Temporomandibular, que utilizaram laser de baixa potência e avaliaram a mastigação e os movimentos mandibulares.

**Análise dos dados:**

Inicialmente realizou-se a leitura dos títulos e resumos de todos os estudos encontrados. Em seguida, apenas os estudos selecionados na primeira etapa foram lidos na íntegra e avaliados quanto à elegibilidade. Após a seleção, foram analisadas as características dos estudos incluídos, bem como a sua qualidade metodológica e da evidência. Na metanálise, a média da amplitude de abertura de boca foi considerada como medida de efeito da intervenção.

**Resultados:**

Verificou-se que os dez artigos incluídos apresentaram resultados muito distintos entre si, principalmente com relação à amplitude de abertura de boca, sendo a mastigação avaliada em apenas um deles. A maioria dos estudos apresentou alto risco de viés, demonstrando uma baixa qualidade metodológica. Considerando os seis estudos incluídos na metanálise, foram identificados resultados significativamente superiores para a fotobiomodulação.

**Conclusão:**

Devido à escassez na literatura, não há evidências suficientes para os efeitos da fotobiomodulação com laser de baixa potência na mastigação. Já nos movimentos mandibulares, notou-se que essa intervenção apresentou resultados significativos, principalmente para o desfecho de amplitude de abertura de boca.

## INTRODUÇÃO

A Disfunção Temporomandibular (DTM) pode ser definida como um conjunto de distúrbios que envolvem os músculos mastigatórios, a articulação temporomandibular (ATM) e estruturas associadas^(1)^. Existem diversas causas para essa disfunção, dentre elas estão os fatores de pré-disposição, precursores e atenuadores, como hábitos deletérios, alterações oclusais, desequilíbrio côndilo-disco, estresse e ansiedade^(2)^. Sendo que, de acordo com a etiologia e os sintomas apresentados pelo paciente, essa desordem pode ser classificada em muscular, articular ou mista^(2)^.

Os sintomas mais comuns dessa desordem são os ruídos articulares (crepitação e estalido), otalgias, zumbido, dores na região da cabeça e pescoço, cefaleia, hiperfunção ou hipofunção da musculatura mastigatória, sensibilidade dentária, desvios mandibulares, redução na abertura de boca, prejuízos no sono, alterações emocionais, diminuindo assim a qualidade de vida dos indivíduos^(3,4)^.

Essa patologia tem crescido de forma significativa, atingindo mais mulheres do que homens, com maior ocorrência na idade entre 20 e 50 anos^(5)^. Por ter uma etiologia multifatorial, o tratamento deve ser realizado de acordo com os sinais e sintomas apresentados pelo paciente, sempre realizando as orientações adequadas, e considerando que a redução de alguns hábitos pode contribuir para a intervenção^(2)^.

Os tratamentos utilizados contemplam procedimentos menos invasivos ou não invasivos, como a terapia medicamentosa, terapia miofuncional orofacial (TMO), tratamento psicológico, placas interoclusais, acupuntura, eletroestimulação, viscossuplementação, ultrassom terapêutico e a laserterapia; como também, os mais invasivos, no caso de cirurgias^(6)^. A terapia para DTM na área da Fonoaudiologia apresenta-se muito eficaz na reabilitação do sistema mastigatório e dos movimentos mandibulares, promovendo uma funcionalidade muscular mais adequada e equilibrada, utilizando exercícios e técnicas da área de Motricidade Orofacial^(7)^.

O *laser* é uma sigla em inglês que, traduzida para o português, significa amplificação de luz por emissão estimulada de radiação. É mais conhecido como terapia com luz, fototerapia ou fotobiomodulação, sendo considerado um dos métodos terapêuticos mais antigos manipulados pelo homem, podendo ser classificados em dois tipos: lasers de alta potência, que são ablativos; e os de baixa potência, que são terapêuticos^(8)^.

A terapia de fotobiomodulação é um tratamento não medicamentoso, indolor, não invasivo e sem efeitos colaterais, que tem como principais funções a analgésica, anti-inflamatória e de regeneração tecidual. Transforma energia luminosa em energia química, induzindo mudanças metabólicas, energéticas e funcionais, contribuindo assim para a elevação da resistência e da vitalidade das células^(9)^.

Em outras áreas, como a Odontologia e Fisioterapia, que já utilizam o laser como tecnologia terapêutica há mais tempo, existem várias pesquisas que demonstram a evidência científica desse recurso na DTM^(9,10)^. No entanto, de um modo geral, observa-se que os desfechos mais estudados são a ação analgésica e os movimentos mandibulares^(11-20)^.

Nos indivíduos com DTM, a dor e o desconforto podem repercutir negativamente no desempenho das funções estomatognáticas. Em estudo realizado com pacientes com DTM crônica moderada-grave, por meio de avaliação funcional e eletromiográfica, identificou-se uma dificuldade significativamente maior na mastigação; piores escores orofaciais; maior tempo para mastigação livre; um recrutamento menos preciso dos músculos nos lados de trabalho e equilíbrio; redução do índice de mastigação simétrico; e aumento da atividade padronizada durante o teste eletromiográfico, quando comparados às pessoas saudáveis^(21)^.

Nesses indivíduos, os efeitos analgésico e biomodulador do LBP, atuando nos processos álgicos e inflamatórios, podem ajudar na diminuição da dor/desconforto e contribuir para a performance muscular, diminuindo a sensibilidade nos músculos mastigatórios e de outros pontos álgicos. Assim, aliado à intervenção fonoaudiológica, o recurso pode permitir um aumento na amplitude dos movimentos mandibulares, promovendo melhora na função mastigatória e proporcionando uma maior harmonia para o sistema estomatognático^(22)^.

Os estudos secundários que pesquisaram as evidências do LBP na DTM evidenciam a importância da terapia de fotobiomodulação no alívio da dor e na melhora da funcionalidade mandibular, como também investigam os efeitos obtidos com a sua associação a outras intervenções. Ao abordar a funcionalidade, as revisões realizadas até o momento não possuem aprofundamento no assunto e geralmente a consideram como um objetivo secundário^(9,23,24)^.

Nesse sentido, o presente estudo foi elaborado para analisar as evidências disponíveis sobre o uso desse recurso nos movimentos mandibulares e na função mastigatória, aspectos que se integram e são de interesse central na intervenção fonoaudiológica em Motricidade Orofacial, nos casos de DTM. Essa revisão foi escrita com base no *Preferred Reporting Items for Systematic Reviews and Meta-Analyses – PRISMA* ^(25)^ e registrada no *International Prospective Register of Systematic Reviews (PROSPERO)*, sob o número CRD42020187091.

## OBJETIVO

Desta forma, a proposta deste estudo foi realizar uma revisão sistemática sobre as evidências da fotobiomodulação com laser de baixa potência, com o objetivo de investigar os efeitos dessa técnica na função mastigatória e nos movimentos mandibulares em adultos com Disfunção Temporomandibular.

## ESTRATÉGIA DE PESQUISA

A construção da estratégia de busca foi realizada com a orientação de uma bibliotecária, servidora da Instituição de origem, sendo adaptada para cada base de dados, utilizando os seus descritores específicos. Os termos foram selecionados a partir da busca nos descritores *Medical Subject Headings – MeSH* da PubMed e dos *EMTREE Terms* algoritmo da EMBASE, considerando a patologia pesquisada, a intervenção e os desfechos incluídos na revisão.

Optou-se por utilizar uma estratégia de busca simplificada, escolhendo os principais termos indexadores disponíveis no vocabulário (tesauro) das bases de dados, pois com o teste prévio da estratégia de busca, percebeu-se que esses eram suficientes e permitiam a recuperação dos estudos elegíveis.

A busca foi realizada nas bases de dados: PubMed, Lilacs (via Biblioteca Virtual em Saúde), *Web of Science, Cochrane Library*, Embase, Scopus, *Science Direct*, e realizou-se uma pesquisa adicional à literatura cinzenta, no Google Scholar e Open Grey. Também foram analisadas as listas de referências dos artigos incluídos no estudo, assim abrangendo alguma referência adicional que não tenha sido identificada durante a busca nos bancos de dados. Além disso, foi realizada a consulta no Registro Brasileiro de Ensaios Clínicos, na tentativa de obter mais informações acerca dos estudos incluídos e identificar possíveis estudos em processo de andamento. A Tabela 1 apresenta as estratégias de busca utilizadas nas bases de dados.

**Tabela 1:** Estratégias de busca utilizadas nas bases de dados

**Estratégia**	**Base de Dados**
Search: ((“temporomandibular joint disorders”) AND (“low level light therapy”)) AND (mastication)(“temporomandibular joint disorders”[All Fields] AND “low level light therapy”[All Fields]) AND (((((((“masticated”[All Fields] OR “masticates”[All Fields]) OR “masticating”[All Fields]) OR “mastication”[MeSH Terms]) OR “mastication”[All Fields]) OR “masticate”[All Fields]) OR “mastications”[All Fields]) OR “masticator”[All Fields]) OR (((“mandible”[MeSH Terms] OR “mandible”[All Fields]) OR “mandibles”[All Fields]) OR “mandible s”[All Fields])	PubMed
((“temporomandibular joint disorders”) AND “low level light therapy”) AND “mastication” OR “mandible” “([Quick search])- Busca Rápida	Embase
(tw:(“temporomandibular joint disorders”)) AND (tw:(“low level light therapy”)) AND (tw:(mastication)) OR (tw:(mandible)) ([Título, resumo e assunto])	Lilacs
“temporomandibular joint disorder” AND “Low level light therapy” AND mastication OR mandible OR mastication*) ([Todos os campos])	Cochrane Library
ALL= (Temporomandibular Joint disorder* AND Low level laser therapy* AND mastication* OR mandible*) ([Todos os campos])	Web of Science
ALL (“temporomandibular joint disorders”) AND ALL (“low level light therapy”) AND ALL (mastication) OR ALL (mandible)) ([Todos os campos])	Scopus
“temporomandibular joint disorders” AND “low level light therapy” OR “low level laser therapy” AND “mastication” OR “mandible” AND “Randomized Controlled Trial”	Google Acadêmico
“temporomandibular joint disorders” AND “low level light therapy” OR “low level laser therapy” AND “mastication” OR “mandible” AND “Randomized Controlled Trial”	ScienceDirect

Foi realizado o gerenciamento das referências e a remoção dos duplicados por meio do *software online* (EndNote Web). Todas as pesquisas nos bancos de dados foram realizadas entre os dias 18 e 20 de maio de 2020. E foi realizada uma atualização da busca no dia 16 de setembro de 2020.

## CRITÉRIOS DE SELEÇÃO

A questão norteadora para a elaboração dessa revisão foi baseada na estratégia PICOT, sendo P- população – adultos com Disfunção Temporomandibular; I – intervenção – Fotobiomodulação com Laser de Baixa Potência; C – comparador – Outras intervenções ou ausência de intervenção; O – outcomes – medidas da função mastigatória e/ou movimentos mandibulares; T – tipos de estudos – Ensaios Clínicos Randomizados. Assim, resultou-se na seguinte pergunta: “Quais os efeitos do Laser de Baixa Potência no desempenho da função mastigatória e nos movimentos mandibulares em indivíduos com DTM, comparado a outras intervenções ou à ausência de intervenção?”.

Foram elegíveis artigos originais com delineamento de ensaio clínico randomizado, sem restrições de período de tempo ou idioma. As pesquisas envolveram adultos com idade entre 18 e 60 anos, com diagnóstico clínico de DTM, intervenção com utilização do LBP e que avaliaram a função mastigatória e/ou os movimentos mandibulares. Foram excluídos os artigos com outros delineamentos, com crianças ou idosos, com texto completo indisponível, com presença de outras comorbidades ou tratamentos associados e aplicados simultaneamente ao laser.

Tais aspectos foram selecionados com base na faixa etária utilizada na maioria dos estudos da área, levando em consideração o desenvolvimento do sistema estomatognático e as modificações decorrentes do processo natural de envelhecimento, e como esses aspectos poderiam influenciar na mensuração dos efeitos da intervenção. A presença de outras comorbidades e de outros tratamentos associados simultaneamente à aplicação do LBP também impossibilitariam uma análise mais precisa dos resultados obtidos. E os principais desfechos elegidos se justificam pela sua relevância clínica na terapia fonoaudiológica nos casos de DTM.

## ANÁLISE DOS DADOS

A seleção dos estudos envolveu duas etapas, sendo ambas realizadas pelos mesmos investigadores, de forma independente. Primeiramente, foi realizada a leitura dos títulos e resumos de todos os estudos encontrados, com exclusão daqueles que não se adequavam aos critérios de elegibilidade previamente estabelecidos. Na segunda etapa, foi realizada a leitura dos textos completos. Em ambas as etapas, foi verificada uma forte concordância interavaliadores, por meio do Coeficiente Kappa de Cohen. As discordâncias foram discutidas entre os autores em ambas as fases do processo de revisão e quando não ocorreu o acordo final entre os mesmos, um terceiro revisor envolveu-se no processo, realizando independentemente a leitura dos estudos, bem como o julgamento da elegibilidade.

Na fase de extração dos dados, as informações também foram coletadas de forma independente pelos dois revisores, sendo elaborado um instrumento específico para essa etapa e realizada a conferência dos dados em uma reunião de consenso. Os dados dos artigos selecionados foram tabelados de acordo com algumas características: autor, país, amostra, objetivo, parâmetros de intervenção, uso do RDC/TMD, tipo de intervenção, desfechos, resultados e conclusão. Quando os estudos apresentaram dados incompletos ou ausentes, os revisores entraram em contato por meio do e-mail disponível para correspondência, buscando obter todas as informações pertinentes.

A qualidade metodológica dos estudos incluídos foi avaliada individual e independentemente por dois revisores, de acordo com a ferramenta da Cochrane *Risk-of-bias tool for randomized trials (RoB 2)* ^(26)^. E a análise da qualidade da evidência foi realizada por meio do *Grading of Recommendations Assessment, Development and Evaluation* (GRADE)^(27)^.

Para a metanálise, a medida de efeito da intervenção considerada foi a média da amplitude de abertura da boca, pois verificou-se que era o principal parâmetro utilizado para a avaliação dos movimentos mandibulares, na maioria dos estudos incluídos. Apenas seis estudos apresentaram em seus resultados a média, desvio padrão e o número de participantes de cada grupo, contribuindo diretamente para a síntese. Já na avaliação da função mastigatória, apenas um estudo incluído considerou esse desfecho. A medida utilizada para fazer a metanálise foi a média e o desvio-padrão, por meio do método do inverso da variância, no software estatístico R.

## RESULTADOS

O detalhamento do processo de busca e seleção dos estudos está apresentado na Figura 1.

**Figura 1:** Fluxograma do processo de busca e seleção dos estudos

Legenda: MD = Diferença de médias; SD = Desvio-padrão

Dos dez estudos selecionados, cinco são brasileiros^(11-15)^ e cinco são internacionais^(16-20)^. Dentre os trabalhos nacionais incluídos, quatro são do estado de São Paulo^(11,13-15)^ e um do Rio Grande do Sul^(12)^. Com relação aos demais artigos, três são de Istambul, na Turquia^(16,18,20)^ e dois de Teerã, no Irã^(17,19)^.

O tamanho da amostra teve uma variação entre 15^(12)^ a 82 participantes^(15)^. Quanto ao protocolo utilizado para o diagnóstico da DTM, oito utilizaram o *Research Diagnostic Criteria for Temporomandibular Disorders* RDC/TMD^(11,14-20)^ e dois não apresentaram qual foi o instrumento utilizado para o diagnóstico^(12,13)^.

Sobre a função mastigatória e os movimentos mandibulares, seis estudos tiveram como um dos desfechos a amplitude de abertura da boca isoladamente^(12-14,16,17,19)^, três analisaram os movimentos protrusivos, de abertura e excursão lateral de mandíbula^(11,18,20)^ e, apenas um deles abordou a função mastigatória^(15)^. O detalhamento das características dos estudos incluídos, bem como os seus principais resultados e conclusões está apresentado no Quadro 1.

**Quadro 1:** Características dos estudos incluídos, principais resultados e conclusões

**Autor**	**País**	**Amostra (n)**	**Objetivo**	**RDC/TMD**	**Parâmetros de intervenção**	**Tipo de intervenção**	**Desfechos**	**Resultados**	**Conclusão**
Silva et al.^(11)^	Brasil	45	Avaliar o efeito do LBP em indivíduos com disfunção temporomandibular intra-articular, quantificar e comparar a gravidade dos sinais e sintomas antes, durante e após as aplicações do laser.	Sim	Foram 10 sessões, realizadas duas vezes por semana, durante 5 semanas. A energia do dispositivo durante as aplicações foi de 70 mW, o tempo de aplicação variou por ponto, e o comprimento de onda utilizado foi de 780 nm. Para a dose de 52,5 J/cm^2^, o tempo de aplicação foi 30 segundos e 60 segundos para a emissão de 105,0 J/cm^2^. O laser foi aplicado de forma contínua em 5 pontos condilares, 3 no masseter e 3 no temporal.	Duas dosagens diferentes de Laser e placebo.	Movimentos mandibulares e sintomas dolorosos evocados pela palpação muscular.	A análise de variância mostrou diferenças estatisticamente significantes entre os grupos, com nível de 1% entre as doses, (exceto para as variáveis de protrusão: diferença significativa de 5% e abertura: não significativa) e também entre as avaliações (exceto para variável lateralidade – 5% de diferença estatística.	Este estudo concluiu que o LBP aumentou a amplitude dos movimentos mandibulares.
De Carli et al.^(12)^	Brasil	15	Comparar o uso do LBP e toxina botulínica no tratamento da dor miofascial e se eles modificam a abertura da boca de pacientes com desordem temporomandibular.	Não informado	Foram 7 aplicações, realizadas em intervalos de 48h entre cada aplicação. O comprimento de onda utilizado foi o de 830nm, com dosagem de 80 J/cm^2^ e potência de 100mW. A aplicação foi em 2 pontos do músculo masseter e 1 no temporal.	Laser e Toxina Botulínica tipo A.	Amplitude de abertura da boca e intensidade de dor.	Em relação a amplitude de abertura da boca, não foi encontrada diferença estatística significante entre o grupo Laser e o grupo Toxina, considerando que ambos os grupos não apresentaram aumento significativo durante o tratamento (p = 0,272).	As duas terapias não apresentaram melhora estatisticamente significativa na amplitude de abertura da boca.
Kato et al.^(13)^	Brasil	18	Comparar a eficácia da estimulação elétrica nervosa transcutânea (TENS) e da terapia a laser de baixo nível no tratamento de pacientes com DTM miogênica crônica.	Não informado	Foram realizadas 10 sessões, 3 vezes por semana, durante 4 semanas, com o comprimento de onda de 830 a 904nm, com uma saída de 4J/cm^2^ e potência de 100mW, com movimentos de varreduras, durante 9 minutos em cada lado da face.	Laser e TENS	Amplitude de abertura da boca e intensidade de dor.	Os resultados demonstraram aumento na amplitude de abertura da boca de ambos os grupos (p < 0,05). Houve uma melhora significativa na amplitude de abertura da boca (média inicial de 42,5 mm, sendo 43 mm do grupo Laser e 42 mm no grupo TENS, e média final de 47,4 mm, sendo 47,6 e 47,2 mm nos grupos Laser e TENS, respectivamente. A ANOVA demonstrou que não houve diferença estatisticamente significante entre os grupos, incluindo o efeito imediato (p = 0,860 e p = 0,091, respectivamente). No entanto, foi encontrada diferença de significância entre as sessões (p<0,0010).	Ambas as terapias foram eficazes em reduzir os sinais e sintomas da DTM, mas o efeito imediato não teve significância e o efeito acumulativo pode ter sido responsável por essa melhora.
Kogawa et al.^(14)^	Brasil	19	Avaliar a efetividade do laser de baixa potência e do estímulo muscular microelétrico (MENS) no tratamento de pacientes com disfunções temporomandibulares (DTM).	Sim	A laserterapia foi realizada três vezes por semana, em um total de dez sessões, com comprimento de onda de 830 a 904nm, com saída de 4 joules por cm^2^ e potência de 100mW.	Laser e MENS	Amplitude de abertura da boca, palpação muscular e da ATM; e Escala Visual Analógica (EVA).	Os resultados apresentaram aumento na amplitude de abertura da boca. As médias inicial e final de amplitude para o grupo Laser foram de 46,3 mm e 49,4 mm, respectivamente e do Estimulador miolétrico (MENS) 46,3 mm e 44 mm, respectivamente. Não mostrando diferença significativa entre os grupos.	Concluiu-se que ambas as terapias (Laser e MENS) são eficazes no tratamento da DTM miogênica, mas recomendou-se cautela na análise dos resultados, pelo aspecto auto limitante da DTM. A amplitude de abertura da boca melhorou nos dois grupos.
Machado et al.^(15)^	Brasil	82	Investigar a eficácia da combinação dos exercícios mioterápicos orais (OM) e LBP, comparado ao protocolo de terapia oromiofuncional (TMO), ao tratamento apenas com LBP, e ao placebo combinado aos exercícios. Verificar os efeitos de cada um dos programas imediatamente após o tratamento e no acompanhamento a longo prazo.	Sim	Foram 12 sessões semanalmente durante 60 dias e depois quinzenalmente. Emissão contínua a 780nm de comprimento de onda, 60mW de potência e densidade de energia variando entre 60 +- 1J/cm^2^. Foi aplicado em 5 locais da ATM e nos músculos masseter e temporal.	Laser + exercícios OM; apenas Laser; placebo + OM e protocolo de TMO completo.	Autoavaliação da gravidade da DTM, dor a palpação, intensidade subjetiva da dor, estado miofuncional orofacial (aparência /postura, mobilidade e desempenho das funções estomatognáticas).	Os resultados do protocolo AMIOFE foram mais baixos que os do grupo placebo, não mostrando resultados estatisticamente significativos entre os grupos.	O LBP com os exercícios OM combinados foi mais eficaz do que apenas o LBP isolado, com redução dos sinais e sintomas da DTM e melhora dos movimentos mandibulares.
Öz et al.^(16)^	Turquia	40	Avaliar a eficiência do laser e compará-lo com placas oclusais no tratamento da dor miofacial.	Sim	Foram realizadas 10 sessões, 2 vezes por semana. O comprimento de onda utilizado foi o infravermelho (820nm), com dosagem de 3J/cm^2^, uma potência de saída de 300 mW e duração de 10s em cada ponto. O laser foi aplicado de forma precisa e contínua nos pontos-gatilho.	Laser e placas interoclusais.	Exame funcional, determinação do limiar de dor à pressão (LDP) e amplitude de abertura da boca.	Ambos os grupos apresentaram melhoras estatisticamente significativas nos movimentos mandibulares verticais, após o tratamento e não foram identificadas diferenças significativas entre os grupos.	O Laser foi tão eficaz quanto as placas interoclusais, podendo ser uma alternativa como tratamento, com base na sua natureza não invasiva e não farmacológica.
Seifi et al.^(17)^	Irã	40	Avaliar o efeito da terapia com LBP e da TENS nas DTM.	Sim	Foram 4 sessões de meia hora por semana. O comprimento de onda utilizado foi de 810nm, com uma potência contínua de 0,5W, durante 60 s.	Laser, placebo do Laser, TENS e placebo da TENS.	Sensibilidade da musculatura mastigatória e amplitude de abertura da boca.	A amplitude de abertura da boca melhorou com o tempo, no entanto, não se manteve após 1 mês em relação a linha de base (p=0,192). Não houve diferenças significativas entre os grupos LBP e TENS (p=0,820) em todas as etapas. Não foi encontrada nenhuma diferença estatística significante entre os placebos (p=0,738). A variável de abertura da boca nos grupos de Laser e TENS foi significante e maior que nos grupos placebos (p=0,002), mas não no seguimento de 1 mês (p=0,692).	Os efeitos do Laser e da TENS podem melhorar os sinais e sintomas da DTM em curto prazo, podendo ser modalidades físicas de alternativas complementares e suplementares na DTM.
Gökçen-Röhlig et al.^(18)^	Turquia	40	Investigar a eficácia do LBP com 820 nm, 3 J/cm^2^, 300 mW potência de saída no tratamento de DTM de origem muscular.	Sim	Foram 10 sessões de laser, realizadas diariamente, durante 3 semanas. O comprimento de onda utilizado foi o infravermelho (820nm), com dose de 8J/cm^2^ e potência de 300mW, por 10 s. A aplicação foi realizada de forma contínua, em 3 pontos do músculo masseter, 1 no temporal e 1 no esternocleidomastoideo.	Laser e placebo	Limiar de dor por pressão (LDP), escala visual analógica (EVA) e mobilidade da mandíbula.	O grupo Laser demonstrou melhorias estatisticamente significativas nos movimentos mandibulares verticais e excursões laterais.	Os efeitos do Laser são superiores aos resultados do grupo placebo.
Madani et al.^(19)^	Irã	45	Investigar a eficácia do LBP no tratamento da osteoartrite e DTM	Sim	Foram 12 sessões, realizadas 3 vezes por semana, durante 4 semanas. O laser foi aplicado em forma de contato, com potência de 50 mW, 6J por ponto, 3,4J/cm^2^ em 4 pontos da ATM, 3 do masseter, 3 do temporal e na inserção do músculo pterigoideo interno.	Laser, Laser acupuntura e placebo	Grau de dor, amplitude de abertura da boca e ruídos articulares.	Após 12 sessões de aplicação do Laser, a amplitude média de abertura da boca aumentou de 29,2mm para 31,7mm (aumento de 8%) no grupo Laser e de 23,5mm para 24,7mm (melhora de 5%) no tratamento com placebo. A análise não mostrou diferença estatística significante entre os grupos de estudo ou entre os diferentes tempos de avaliação em cada grupo.	Os grupos Laser e placebo não apresentaram melhora significativa em relação à amplitude de abertura da boca.
Sancakli et al.^(20)^	Turquia	30	Avaliar o efeito da aplicação do LBP nos maiores pontos de dor em pacientes com dor muscular mastigatória crônica (dor miofascial).	Sim	Foram 12 sessões, realizadas 3 vezes por semana, durante 1 mês. O comprimento de onda utilizado foi o infravermelho (820nm), com dose de 3J/cm^2^ e potência de saída de 300 mW por 10 s. A aplicação foi realizada nos maiores pontos de dor e em 3 pontos dos músculos masseter e temporal.	Laser nos maiores pontos de dor, e em pontos pré-determinados.	Mobilidade de mandíbula (amplitude, excursões laterais, protusão), LDP e medida da pressão, e medida subjetiva da dor.	Os movimentos mandibulares verticais, excursões laterais e protrusão melhoraram significativamente nos grupos de LBP.	O LBP apresentou efeitos positivos em relação aos movimentos mandibulares, devido aos seus efeitos analgésicos e miorrelaxantes. Não houve diferença entre a aplicação nos pontos dolorosos e nos pontos pré-determinados.

Dos estudos que apresentaram resultados significativos com relação à amplitude de movimento mandibular, verificou-se que quanto maior a dose utilizada, os efeitos eram imediatos e mais expressivos, com mensurações entre a primeira, quinta, décima, décima segunda sessões e até um mês após a intervenção com laser.

Alguns estudos apontaram que os resultados de amplitude de abertura da boca não foram estatisticamente significativos entre os grupos^(12-14,16,17,19)^. Com relação aos movimentos verticais, de excursões laterais e protrusão, três artigos^(11,18,20)^ mostraram resultados estatisticamente significantes.

Apenas um estudo incluído avaliou a função mastigatória, demonstrando que a terapia de fotobiomodulação com LBP combinada com os exercícios oromiofuncionais é mais eficaz do que apenas o LBP isolado, com redução dos sinais e sintomas da DTM e melhora dos movimentos mandibulares. Nesse estudo, os resultados dos escores gerais de mobilidade e de funções indicaram resultados inferiores no grupo tratado apenas com a laserterapia, com diferenças significativas entre os grupos^(15)^.

Dos dez estudos incluídos, nove deles foram agrupados para a análise quantitativa dos resultados, por apresentarem a medida de amplitude de abertura da boca, sendo que, destes, apenas seis puderam ser utilizados na metanálise. Verificou-se que os estudos eram muito distintos entre si, principalmente com relação à amplitude oral máxima.

Na análise quantitativa, pode-se observar que o losango do final do gráfico reflete a combinação dos resultados. Como ele está do lado direito e não tocou o eixo, o tratamento com grupo experimental é melhor, ou seja, mostrou resultados significativos. Já o valor da coluna diferença de médias (MD), revela que o grupo experimental foi melhor 2,78 pontos numa escala de 0 a 100 nos modelos aleatórios. Com relação a heterogeneidade entre os estudos, I^2^ = 60%, indicando heterogeneidade moderada. A Figura 2 apresenta o detalhamento da síntese quantitativa.

**Figura 2:** Forest Plot da metanálise dos estudos

Os estudos incluídos apresentaram bastante heterogeneidade metodológica. Cinco artigos^(11,13,14,17,18)^ apresentaram uma classificação geral de alto risco de viés, dois foram classificados com algumas preocupações^(16,20)^ e três considerados de baixo risco^(12,15,19)^ pela avaliação de qualidade aplicada.

As principais limitações metodológicas encontradas nos estudos foram relativas às informações não contidas sobre a geração de sequência aleatória, ocultação de alocação e cegamento de participantes, conforme exposto na Figura 3.

**Figura 3:** Classificação geral e categorização de qualidade dos estudos incluídos

Como a revisão utiliza desfechos provenientes de ensaios clínicos randomizados, a avaliação da qualidade da evidência foi iniciada com pontuação máxima, sendo posteriormente rebaixada em alguns parâmetros, conforme o Quadro 2.

**Quadro 2:** Qualidade da evidência (GRADE)

**Sumário de Resultados**						
**Fotobiomodulação com LBP comparada ao Placebo ou outras intervenções para Disfunção Temporomandibular**						
**Paciente ou população**: Disfunção Temporomandibular **Contexto**: Movimentos mandibulares e função mastigatória **Intervenção**: Fotobiomodulação com LBP **Comparação**: Placebo ou outras intervenções						
Desfecho Nº de participantes (estudos)	Efeito relativo (IC 95%)	**Efeitos absolutos potenciais (IC 95%)**			Certeza	O que acontece
		Sem Fotobiomodulação com LBP	Com Fotobiomodulação com LBP	**Diferença**		
Movimentos mandibulares avaliado com: Abertura oral Nº de participantes: 160 (6 ECRs)	-	A média dos movimentos mandibulares foi de **37,25** mm	A média dos movimentos mandibulares foi de **40,03** mm	DM **2.78 mm mais alto** (1.12 mais alto para 4.44 mais alto)	⨁◯◯◯ MUITO BAIXA ^a,b,c^	A Fotobiomodulação com LBP pode aumentar/ter pouco ou nenhum efeito nos movimentos mandibulares mas as evidências são muito incertas.
Função mastigatória avaliado com: Protocolo AMIOFE Nº de participantes: 39 (1 ECR)	O estudo apresentou apenas a análise dos escores totais das funções estomatognáticas, identificando que o grupo de intervenção com Fotobiomodulação com LBP não obteve resultados significativos após o tratamento. Já na comparação entre os diversos grupos de intervenção, foram identificados resultados significativos.				⨁⨁⨁⨁ ALTA	O estudo apresentou alta qualidade metodológica, entretanto seria necessária uma análise específica da mastigação, bem como a combinação com outros estudos. Desse modo, não existem evidências suficientes para os efeitos da Fotobiomodulação com LBP na função mastigatória.
**Graus de evidência do GRADE Working Group** **Alta certeza**: estamos muito confiantes de que o verdadeiro efeito está próximo ao da estimativa do efeito **Certeza moderada**: estamos moderadamente confiantes na estimativa do efeito: o verdadeiro efeito provavelmente estará próximo da estimativa do efeito, mas existe a possibilidade de que seja substancialmente diferente **Baixa certeza**: Nossa confiança na estimativa do efeito é limitada: O verdadeiro efeito pode ser substancialmente diferente da estimativa do efeito **Certeza muito baixa**: temos muito pouca confiança na estimativa do efeito: o efeito verdadeiro provavelmente será substancialmente diferente da estimativa do efeito						
**Explicações** a. Os estudos apresentaram muitas limitações metodológicas com relação ao desfecho avaliado. Dos seis estudos incluídos, três deles apresentaram alto risco de viés, um com risco incerto e dois com baixo risco, contribuindo para rebaixar a qualidade da evidência. Alguns dos aspectos identificados foram: falta de sigilo da alocação dos participantes, falta de cegamento e falta de informações sobre as perdas de seguimento. b. Comparando os estudos entre si, identificou-se a presença de inconsistência, pois para um mesmo desfecho, foram utilizadas diversas metodologias, com parâmetros de intervenção e resultados distintos, implicando em uma heterogeneidade considerável, também verificada na análise estatística, rebaixando a qualidade da evidência encontrada. c. Alguns estudos apresentaram imprecisão na amplitude do intervalo de confiança de 95%, diminuindo a confiança nas estimativas do efeito.						

Legenda: IC = intervalo de confiança; DM = diferença de médias

Os resultados evidenciaram que a comparação do laser com as intervenções de toxina botulínica tipo A^(12)^ e estímulo muscular microelétrico (MENS)^(14)^ não apresentou resultados significativos com relação ao desfecho de movimentos mandibulares. Já ao comparar a estimulação elétrica nervosa transcutânea (TENS) e o LBP, em um estudo mostrou-se eficácia de ambas as terapias, havendo diferenças entre os grupos apenas no efeito acumulativo^(13)^; e no outro, não houve diferenças significativas entre os grupos LBP e TENS, em todas as etapas^(17)^.

Quando houve comparação entre duas modalidades de LBP diferentes ou com o grupo placebo^(11,18,19)^, percebeu-se que os resultados foram significativos e superiores para os grupos que receberam a intervenção. No trabalho que comparou o laser com a TMO^(15)^, identificou-se grandes resultados desta terapia de forma isolada, mas não foi combinada à terapia de LBP, podendo ser uma possível alternativa para que se obtenham resultados mais significativos.

Verificou-se que um importante aspecto a ser considerado diz respeito à dose utilizada, pois alguns estudos utilizaram uma dose baixa, entre 1,5J – 3J, que para os objetivos propostos, talvez não trouxesse significância, sendo necessário uma dose maior. Considerando tais resultados, percebeu-se uma grande inconsistência entre os estudos incluídos, bem como a presença de falhas metodológicas, fazendo com que cinco dos estudos ficassem com alto risco de viés, abaixando assim a qualidade da evidência e a não confiança dos mesmos.

Também houve grande variabilidade no protocolo de laser utilizado em cada estudo. Com relação ao número de sessões, os estudos mostraram que há um equilíbrio entre 10 a 12 sessões, como preconiza a literatura, pois é considerado o número de sessões adequado para que se obtenham resultados significativos. Levando em consideração a periodicidade das sessões, observou-se uma variação entre uma vez por semana, todos os dias durante quatro semanas, cinco semanas ou quinzenalmente.

Com relação ao comprimento de onda, existiu uma variação de 780nm a 904nm, identificando assim que todos os estudos utilizaram o comprimento de onda infravermelho. A maior dissemelhança entre os estudos foi quanto à dose aplicada, que variou entre 1,5 J/cm^2^ a 105,0 J/cm^2^, a depender do equipamento que foi utilizado. Assim, os estudos revelam o quanto são heterogêneos, sendo necessário que, nos próximos ensaios clínicos com laser, sejam escolhidos protocolos mais homogêneos e com um maior rigor metodológico, para que os resultados possam ter uma evidência mais confiável.

Dos 10 estudos, cinco são brasileiros^(11-15)^, mostrando que o Brasil tem forte publicação na área da Fotobiomodulação e DTM, como também, três estudos integram o mesmo grupo de pesquisa^(13-15)^. Quase todos ensaios clínicos são de áreas como a Fisioterapia ou Odontologia e, apenas um artigo^(15)^ traz uma fonoaudióloga entre os autores, o qual também é o único que analisa a função mastigatória. Isso pode ocorrer devido a essas ciências utilizarem o laser há mais tempo e na Fonoaudiologia a regulamentação de seu uso ocorreu apenas no ano de 2019, por meio da Resolução de nº 541^(28)^, associada a uma recente aplicação desse recurso na prática clínica.

Tais aspectos demonstram que esses profissionais precisam executar mais pesquisas sobre função mastigatória e movimentos mandibulares, já que são fundamentais na terapia com DTM. Atualmente dispomos de achados clínicos positivos, mas ainda necessitamos de maiores evidências científicas para recomendar a escolha terapêutica e a tomada de decisão pela utilização desse recurso em detrimento ou associado aos outros já disponíveis na área.

Considerando que o laser pode atuar estimulando ou inibindo a resposta do tecido, nos indivíduos com DTM, esse recurso pode favorecer o desenvolvimento das funções alteradas, inclusive a mastigação, que possui um impacto considerável nessa patologia^(22)^. É importante salientar que, na clínica fonoaudiológica, essa tecnologia não deve ser utilizada para substituir terapias consistentes e de alta relevância na área, mas sim como uma intervenção coadjuvante e alternativa, a fim de promover uma aceleração no processo de tratamento. Assim, é necessária uma intervenção direcionada e individualizada, que envolva uma integração entre as diversas abordagens envolvidas no cuidado aos indivíduos com DTM e considere os distintos aspectos fonoaudiológicos e odontológicos envolvidos na reabilitação dessa função.

Diante do exposto, algumas implicações clínicas decorrentes desse estudo se destacam. É bastante evidente a necessidade da elaboração de protocolos de intervenção, que tragam uma maior padronização de parâmetros importantes, como a dosimetria, o número e a periodicidade de sessões, na busca por resultados terapêuticos efetivos.

Essa revisão apresenta algumas contribuições ao apontar os principais parâmetros considerados atualmente pela literatura científica da área e os resultados com eles obtidos. Também contribui ao evidenciar os efeitos obtidos com o laser comparados aos de outras intervenções, facilitando assim a prática clínica dos fonoaudiólogos na área, sobre a escolha da melhor abordagem terapêutica para o objetivo pretendido.

Outro aspecto importante é que a medida mais recorrentemente encontrada na literatura, seja como desfecho principal ou secundário, foi a amplitude de abertura da boca, sendo assim, verificou-se que a medida desse parâmetro é de fundamental importância para a quantificação dos resultados obtidos na intervenção com o laser. Contudo, é imprescindível uma avaliação mais robusta, que considere critérios mais amplos na análise dos diversos movimentos mandibulares.

Nesta revisão sistemática, algumas limitações precisam ser apontadas. Foi encontrada uma considerável variabilidade na análise dos estudos incluídos. Isso pode ocorrer devido às características dos estudos individuais, que são bem divergentes quanto à metodologia aplicada (tamanho da amostra, tipo de intervenção utilizada, potência, dose de energia, tempo de aplicação, etc.). Dessa forma, mesmo que existam algunss efeitos positivos quanto a eficácia do laser na DTM, os diversos parâmetros metodológicos utilizados interferem nas conclusões obtidas nos estudos individuais, que apresentam resultados distintos e conflitantes entre si.

Além das divergências metodológicas encontradas entre os estudos, também foi verificada uma baixa qualidade da evidência, com a presença de um viés considerável na maioria dos estudos. Destaca-se também os dados ausentes nos estudos e a dificuldade em conseguir as informações para uma síntese quantitativa que incluísse todos os resultados, permitindo uma análise mais ampla.

Diante disso, neste estudo verificou-se que a fotobiomodulação com LBP não trouxe evidências quanto ao efeito do LBP para o benefício da função mastigatória, mas demonstrou os efeitos benéficos para o aumento da amplitude dos movimentos mandibulares. A terapia com LBP promoveu impactos positivos no aumento da amplitude de abertura da boca, com melhores resultados quando comparada às outras intervenções ou à ausência de tratamento, conforme demonstrado na metanálise.

Destaca-se a necessidade da elaboração de novos ensaios clínicos, com protocolos mais homogêneos e com alta qualidade, para que novas abordagens clínicas e evidências científicas surjam e possam ser replicadas, principalmente na área da Fonoaudiologia em que se apresentou uma escassez de estudos com ênfase na função mastigatória.

## CONCLUSÃO

Tendo em vista que apenas um estudo abordou a função mastigatória, verificou-se que há uma escassez na literatura quanto à análise dessa variável. Portanto, não há informações suficientes para realizar uma análise quanto aos efeitos da terapia de fotobiomodulação com LBP nessa função. Com relação aos movimentos mandibulares, as pesquisas são muito variáveis quanto aos parâmetros metodológicos utilizados e desfechos pretendidos. Nos grupos de intervenção, a Fotobiomodulação com LBP apresentou resultados significativos, evidenciados na síntese quantitativa do desfecho principal de amplitude de abertura da boca.
